# Neural processes of inhibitory control in American Indian peoples are associated with reduced mental health problems

**DOI:** 10.1093/scan/nsac045

**Published:** 2022-07-08

**Authors:** Evan J White, Mara J Demuth, Mariah Nacke, Namik Kirlic, Rayus Kuplicki, Philip A Spechler, Timothy J McDermott, Danielle C DeVille, Jennifer L Stewart, John Lowe, Martin P Paulus, Robin L Aupperle

**Affiliations:** Laureate Institute for Brain Research, Tulsa, OK 74136, USA; Oxley School of Community Medicine, University of Tulsa, Tulsa, OK 74119, USA; Laureate Institute for Brain Research, Tulsa, OK 74136, USA; Laureate Institute for Brain Research, Tulsa, OK 74136, USA; Laureate Institute for Brain Research, Tulsa, OK 74136, USA; Laureate Institute for Brain Research, Tulsa, OK 74136, USA; Laureate Institute for Brain Research, Tulsa, OK 74136, USA; Laureate Institute for Brain Research, Tulsa, OK 74136, USA; Department of Psychology, University of Tulsa, Tulsa, OK 74104, USA; Laureate Institute for Brain Research, Tulsa, OK 74136, USA; Department of Psychology, University of Tulsa, Tulsa, OK 74104, USA; Laureate Institute for Brain Research, Tulsa, OK 74136, USA; Oxley School of Community Medicine, University of Tulsa, Tulsa, OK 74119, USA; School of Nursing, University of Texas at Austin, Austin, TX 78712, USA; Laureate Institute for Brain Research, Tulsa, OK 74136, USA; Oxley School of Community Medicine, University of Tulsa, Tulsa, OK 74119, USA; Laureate Institute for Brain Research, Tulsa, OK 74136, USA; Oxley School of Community Medicine, University of Tulsa, Tulsa, OK 74119, USA

**Keywords:** American Indians, suicide risk, inhibitory control, fMRI, substance use, protective factors

## Abstract

American Indians (AI) experience disproportionately high prevalence of suicide and substance use disorders (SUD). However, accounting for risk burden (e.g. historical trauma and discrimination), the likelihood of mental health disorders or SUD is similar or decreased compared with the broader population. Such findings have spurred psychological research examining the protective factors, but no studies have investigated its potential neural mechanisms. Inhibitory control is one of the potential neurobehavioral construct with demonstrated protective effects, but has not been examined in neuroimaging studies with AI populations specifically. We examined the incidence of suicidal thoughts and behaviors (STB) and SUD among AI (*n *= 76) and propensity matched (sex, age, income, IQ proxy and trauma exposure) non-Hispanic White (NHW) participants (*n = *76). Among the AI sample, functional magnetic resonance imaging (fMRI) data recorded during the stop-signal task (SST) was examined in relation to STB and SUDs. AIs relative to NHW subjects displayed lower incidence of STB. AIs with no reported STBs showed greater activity in executive control regions during the SST compared with AI who endorsed STB. AI without SUD demonstrated lower activity relative to those individual reporting SUD. Results are consistent with a growing body of literature demonstrating the high level of risk burden driving disparate prevalence of mental health concerns in AI. Furthermore, differential activation during inhibitory control processing in AI individuals without STB may represent a neural mechanism of protective effects against mental health problems in AI. Future research is needed to elucidate sociocultural factors contributing protection against mental health outcomes in AIs and further delineate neural mechanisms with respect to specific concerns (e.g. SUD *vs* STB).

## Background

Previous studies reported that American Indian (AI)^1^ populations suffer from disproportionately high prevalence of suicide and substance use disorders (SUD) (Beals, 2005; Brave Heart *et al.*, 2016). Alarmingly, AI populations have demonstrated the highest increase in suicide rates of any ethnic group from 1999 to 2017, 139% for females and 71% for males (Curtin and Hedegaard, 2019). However, disparate prevalence has often been noted in the literature without consideration of factors, which likely drive higher levels of risk in these communities; specifically, it has been noted that systemic racism confers disproportionate health risks in particular racial categories (Jones, 2000). Among AI communities, many disproportionate risk factors, such as historical trauma, discrimination and trauma exposure contribute to increased risk for mental health concerns (Alcántara and Gone, 2007; Walls *et al.*, 2021). Appropriate contextualization of research in health and mental health among marginalized populations is a critical consideration in this field due to the potential for harm to communities from unintended biases, misinterpretations and promulgation of stereotypes and prejudice (Beals *et al.*, 2009; Jordan *et al.*, 2021).

When researchers contextualize mental health concerns with respect to risk factors (e.g. trauma exposure and socioeconomic status), disparate prevalence has been demonstrated to vanish (Beals *et al.*, 2002). In some work, AI individuals actually show lower prevalence of certain conditions (e.g. depression, anxiety; Beals, 2005). For example, the prevalence of post-traumatic stress disorder (PTSD) among AIs may be inflated due to higher prevalence of trauma exposure (Robin *et al.*, 1997; Beals *et al.*, 2002; Manson *et al.*, 2005). Thus, mental health disparities among AIs are likely driven by increased risk burden (Beals, 2005; Evans-Campbell *et al.*, 2006; King *et al.*, 2009; Kading *et al.*, 2015). However, contextualizing mental health concerns with respect to particular risk factors is an underdeveloped area of research particularly in the context of SUD.

There is a burgeoning body of previous and ongoing work, which has focused on mental health research and the development of community programs to mitigate risk burden and promote well-being among AI populations (Les Whitbeck *et al.*, 2004; Blue Bird Jernigan *et al.*, 2015; Walters *et al.*, 2019). Furthermore, recent research in AI mental health has focused on strength-based approaches for understanding the protective and resilience factors (Stumblingbear-Riddle and Romans, 2012; Wexler, 2014; Elm *et al.*, 2016; Oré *et al.*, 2016; Teufel-Shone *et al.*, 2018), which is a significant gap in quantitative research in AI populations. Specific calls related to strengths-based research and protective factors in AIs highlight the need for understanding the mechanisms of protective effects, multi-level interaction and consideration of unintended impacts among other recommendations (Allen *et al.*, 2022).

Clinical neuroscience techniques hold significant promise for extending the level of analysis in mental health research in AI and characterizing mechanisms of risk and protective factors. Notably, broader calls for clinical neuroscience work aim at integrating knowledge of brain and nervous system function from basic science research into an understanding of etiology and maintenance of mental disorders and substance use with an ultimate goal of informing intervention and prevention efforts (e.g. Insel and Quirion, 2005; NIDA, 2010; NIMH, 2021). Unfortunately, very little research has investigated psychological and neural mechanisms underlying the risk for protection against mental health problems within AI populations. In fact, AI populations are largely absent in mental health neuroscience research in general (e.g. neuroimaging and genetics research). This is problematic as advances in clinical neuroscience identified in the general population may not be generalized to AI communities; furthermore, there may be unique aspects of AI populations that confer protection against mental health difficulties that neuroscience could be helpful in further delineating. This underscores the need to have clinical neuroscience research within AI populations to inform our understanding of potential common and unique protective and risk factors. Furthermore, such work represents an intersection of the cutting edge of both AI mental health research, which calls for understanding the mechanisms and multi-level interactions (Allen *et al.*, 2022) and clinical neuroscience priorities in increasing diversity and understanding the environmental influences on risk for mental health conditions (NIMH, 2021).

The dearth of neuroscience research among AIs is a critical gap in the overarching goal of reducing mental health disparities among AI and there are numerous potential functional domains that may be relevant for the study of neuroscientific indicators of adaptive functioning in AI populations. A prudent first step is to examine well-established adaptive neurocognitive functions among AI samples to determine the generalizability of findings within this population and establish associations with mental health concerns. Inhibitory control is a cognitive function that serves to prevent and regulate impulsive behaviors (Logan *et al.*, 1997) and prepotent behavioral responses during both neutral and emotional situations (Diamond, 2013). Higher levels of inhibitory control are associated with lower levels of multiple forms of psychopathology across behavioral and functional brain studies (Aupperle *et al.*, 2012; McTeague *et al.*, 2016; White and Grant, 2017). Neural correlates of adaptive inhibitory control have also been associated with decreased risk for SUD (Holmes *et al.*, 2016) and increased ability to regulate craving for substances (Kober *et al.*, 2010). Furthermore, deficits in inhibitory control are associated with increased risk of suicidal behavior (Nock *et al.*, 2010; Jollant *et al.*, 2011). Notably, specific brain regions associated with inhibitory control are sensitive to functional context and measurement strategy (e.g. task design). The dorsolateral prefrontal cortex (dlPFC), in particular, has been implicated in inhibitory control in emotional contexts (Goldstein *et al.*, 2007). Additionally, a well-established empirical paradigm for indexing inhibitory control (e.g. SST; Matthews *et al.*, 2005) indicates blood-oxygenation level dependent (BOLD) signal increases in the dlPFC and inferior frontal gyrus (IFG) under inhibitory control demands. Both the IFG and dlPFC have also been implicated in research examining response inhibition deficits associated with SUD (see Spechler *et al.*, 2016). Thus, inhibitory control as indexed by BOLD signal response in IFG and dlPFC during SST is an ideal candidate for the initial exploration of a potential mechanism for adaptive cognitive function that may be inversely related to mental health concerns among AI populations.

The intention of the current study was to explore potential adaptive functioning mechanisms and provide a foundation for a strength-based neuroscientific perspective of mental health among AI populations. There is precedent and potential for stigma and inadvertent harm that can come from research on mental health and substance use conditions affecting Indigenous communities (e.g. Beals *et al.*, 2009; Drabiak-Syed, 2010). Thus, aim 1 of the current study examines the incidence of suicidal thoughts and behaviors (STB) reported during clinical interview and SUD across the lifespan among a sample of AI individuals in comparison to a non-Hispanic white (NHW) sample, to demonstrate that sociodemographic and risk factors (i.e. trauma-exposure) likely account for potential disparities present in this sample consistent with previous research (e.g. Beals *et al.*, 2002). Aim 2 of the current study is to delineate difference in neural markers of inhibitory control for AI individuals with and without STB and for those with *vs* without SUD. Notably, aim 2 is conducted only within the AI group as the intention of the study is to establish potential neural mechanisms of adaptive cognitive function relative to mental health concern in this population. Furthermore, comparisons across racial groups are inconsistent with the goals of the current work as race is not appropriately conceptualized as a causal (Holland, 2003) or biological (Templeton, 2013) variable and may be better conceptualized as epi-phenomenological considering the differential exposure to modifiable factors (e.g. income, access to healthcare, education and discrimination) across racial categories. Regarding the first aim, we hypothesized that when propensity matching for sociodemographic and risk factors AIs would demonstrate equivalent and or lower incidence of STB and SUD than NHW, consistent with previous research. With respect to aim 2, we hypothesized AI individuals with either no STB or without SUD would exhibit higher levels of activation in inhibitory control networks (Spechler *et al.*, 2016) (i.e. dlPFC and IFG) during response inhibition in the SST compared with AI individuals with STB and/or SUD.

## Methods and materials

### Participants

Participants for the current study were drawn from the first 500 participants in the baseline assessment of the Tulsa-1000 (T1000) study (Victor *et al.*, 2018). The total T1000 sample is divided into two halves (*n *= 500/sample), the first half for exploratory studies and the second half with controlled access is for confirmatory, replication and preregistered studies. The current investigation falls in the former category. The T1000 study recruited treatment seeking adults (age 18–55) from the Tulsa community and local treatment facilities with mood, anxiety, substance use and eating disorders, as well as healthy comparison participants, in order to identify neural, behavioral, self-report, physiological and biological variables derived from blood-based analytes to identify factors with potential clinical utility. Screening for anxiety, mood, substance use and eating disorders was based on Patient Health Questionnaire-9 score ≥ 10, Overall Anxiety Sensitivity and Impairment Scale ≥ 8; Drug Abuse Screening Test-10 > 2 Sick, Control, One, Fat, Food Questionnaire score ≥ 2. Full screening procedures and inclusion/exclusion criteria are reported in the open access T1000 protocol paper (Victor *et al.*, 2018). This study was reviewed and approved by the Western Institutional Review Board. All participants provided written informed consent prior to participation, in accordance with the Declaration of Helsinki, and were compensated for participation (ClinicalTrials.gov identifier: #NCT02450240).

Participants in the current analyses self-identified their race and ethnicity. Those who self-identified as AI to any degree were included in the AI group (*n *= 89) and those who identified as NHW were included in the comparison group (*n *= 348) for a total initial sample of (*n *= 437). Participants were administered the MINI International Neuropsychiatric Interview (6.0 or 7.0) (Sheehan and Lecrubier, 2010; Sheehan *et al.*, 2015) by clinical study staff to assess lifetime mental disorders, defined by the Diagnostic and Statistical Manual of Mental Disorders (4th or 5th Edition; American Psychiatric Association, 2000, 2013). Demographic information and screening measure scores are described in Table 1. Thirteen individuals in the AI group were excluded from analyses due to missing data on variables of interest resulting in a sample of (*n* = 76) for analysis of incidence rates. Propensity matching procedures (see Analytic Strategy) generated a matched sample of NHW participants (*n* = 76) from the broader pool of participants. For subsequent fMRI BOLD signal analysis among the AI subsample eight subjects were excluded for poor quality data (see Neuroimaging Data Processing section below) resulting in a final sample size of *n* = 68.

**Table 1.** Demographic and screening data in the full sample for American Indian (AI) and non-Hispanic White (NHW) groups

	AI (*n* = 89)		NHW (*n* = 387)				
	*M*	*SD*	*M*	*SD*	*t*	*df*	*p*
Age (years)	34.65	9.61	34.59	11.07	0.05	156	0.96
Income ($)	28 238.38	32 874.05	54 634.89	79 409.82	−4.57	313	<0.001
PHQ	8.27	5.59	9.52	6.68	−1.82	162	0.07
DAST	4.20	3.85	2.46	3.54	3.89	130	<0.001
OASIS	7.17	5.01	7.57	4.60	−0.69	130	0.49
SCOFF	0.63	1.02	0.97	1.27	−2.67	168	0.008
	Frequency		Frequency				
Education (categories)					*Χ* ^2^ = 10.66, *p *= 0.01		
Less than high school	15 (16.67%)		26 (7.51%)				
High school or GED	24 (26.67%)		69 (19.94%)				
Some college	24 (26.67%)		127 (36.71%)				
College degree or higher	27 (30%)		124 (35.83%)				
Sex (female)	52 (57.78%)		229 (65.81%)		*Χ* ^2^ = 2.00, *p *= 0.16		

Note: PHQ = Patient Health Questionnaire-9; DAST = Drug Abuse Screening Test; OASIS = Overall Anxiety Severity and Impairment Scale; SCOFF = ‘SCOFF’ questionnaire screening tool for eating disorders.

### Procedure

Overall procedures included clinical interview and assessment sessions and a neuroimaging session completed within a 2-week period. Only details relevant to the current analyses are presented here, but the full protocols of the parent project are available (Victor *et al.*, 2018).

#### Clinical interview and measures

The MINI clinical interview was administered by trained study staff and during this session participants provided self-reported information regarding demographics (i.e. age, income, race and ethnicity), trauma exposure and completed the Wide Range Achievement Test (Wilkinson and Robertson, 2006). Trauma exposure was assessed using Traumatic Events Questionnaire (Vrana and Lauterbach, 1994) and the Childhood Trauma Questionnaire (Bernstein *et al.*, 2003). STB was assessed by the MINI interview as indicated on the suicidality module with questions regarding suicidal ideation, intent, plan and previous suicidal behaviors within the past month. Specifically, individuals who met criteria for any level of STB (i.e. low, moderate and high) were included in the STB group and those who did not were included in the no-STB group. The presence of SUD was established based on any substance use disorder diagnosis within the year prior to participation integrating reporting information from the MINI and the Tulsa Life Chart (Aupperle *et al.*, 2020), with life time substance use verified by the Customary Drinking and Drug Use Record (Brown *et al.*, 1998). See supplementary information for frequency of various current substances of choice present in the matched samples (Table S1), number of individuals with SUD diagnosed by the MINI or Tulsa Life Chart or both and those without SUD (Table S2).

#### Neuroimaging session

Participants completed 288 trials of the SST (Matthews *et al.*, 2005) over approximately 8 min and 32 s during fMRI. In this task, participants were asked to respond to an ‘X’ and ‘O’ with either a right or left button press, but on 25% of the trials (*n *= 72), an auditory tone and color change (i.e. ‘stop-signal’) indicated they should not respond. Notably, this task was previously examined in a portion of the T1000 sample (*n* =121) with respect to anxiety, depression and cannabis use (Spechler *et al.*, 2020). Trials were presented in six blocks of 48 trials with 12 s breaks between blocks. Difficulty of the trials was manipulated by delay of stop-signal following target stimuli calibrated to each individual during practice trials. Easy trials having longer delays (i.e. 500, 400 and 300 ms) and hard trials having shorter delays (i.e. 200, 100 and 0 ms) relative to participants mean reaction RT on practice trials. For the current study, response inhibition is indexed as the percent signal change (PSC) in BOLD response on hard compared with easy successful stop trials.

Functional magnetic resonance imaging (fMRI) was acquired during the SST with two identical GE MR750 3 T scanners consisting of contiguous echo-planar imaging (EPI) volumes (39 axial slices, TR/TE = 2000/27 ms, flip angle = 78°, sampling bandwidth = 250 kHz, FOV/slice thickness = 240/2.9 mm, 128 × 128 matrix producing 1.875 × 1.875 × 2.9 mm voxels, 256 volumes, R/L frequency encoding direction, SENSE acceleration factor = 2). Additionally, high-resolution structural images were obtained through a 3D axial T1-weighted magnetization-prepared rapid acquisition with gradient echo sequence (TR/TE = 5/2.0 12 ms, FOV/slice thickness = 240/0.9 mm, flip angle = 8°, sampling bandwidth = 31.25 kHz, 256 × 256 matrix producing 0.938 × 0.938 × 0.9 mm voxels, 186 axial slices, SENSE acceleration factor = 2).

### Neuroimaging data processing

The current study included AI participants with quality fMRI data (*n *= 68). Participants were excluded if they had an average Euclidean norm of the derivatives of the six motion parameters (ENORM) greater than 0.3 across and had significant artifacts on visual inspection, or no SST data available. Neuroimaging data were processed and analyzed using Analysis of Functional Neuroimaging (AFNI; http://anfi.nimh.nih.gov) software (Cox, 2012). The three initial EPI volumes during the run were removed to allow time for signal stabilization and noise adaptation. Then data processing included despiking, correction for slice timing, co-registering to anatomical volumes, correction for motion via affine registration, smoothing (4 × 4 × 4 mm^3^ full width at half maximum) and normalizing to Montreal Neurological Institute standard space (resampled voxel size 2 × 2 × 2 mm^3^). General linear model analyses were employed to model task-related brain activation. Modeling included five task-related regressors: successful easy stops, successful hard stops, unsuccessful easy stops, unsuccessful hard stops and successful go trials. Additionally, nuisance regressors included six motion parameters (roll/pitch/yaw/x/y/z translation) and four baseline polynomials. Any TR with an ENORM greater than 0.3 or an outlier fraction greater than 0.1 (via 3dToutcount) was censored at the regression step. Model fits were estimated from single subject general linear models (i.e. beta coefficients for condition contrasts hard minus easy). Data were then extracted for regions of interest (ROIs) corresponding to brain regions activated in the SST previously (IFG and dlPFC) (Matthews *et al.*, 2005) using the Brainnetome Atlas (BNA) (Fan *et al.*, 2016); specific Brainnetome labels ROIs are reported in supplementary materials; Table S3). ROIs were constructed for the left and right hemisphere separately to account for potential laterality effects. Only voxels with a temporal signal to noise ratio of greater than 50 were included in ROI analyses. Task effect images from the parent sample (Figure S1 and Table S4) and exploratory whole brain analyses in the AI subsample can be found in supplementary material (Figures S2 and S3).

### Analytic strategy

#### Incidence of STB and SUD

Propensity matching was employed to examine incidence of STB and SUD in the current sample, while properly accounting for sociodemographic factors and psychological risk factors. Propensity score matching is used in observational studies to reduce bias introduced by covariates by balancing them across two comparison groups (D’Agostino, 1998). Propensity matching was conducted using the *MatchIt* R program (Ho *et al.*, 2011) following procedural recommendations from Randolph *et al.* (2014). Individuals who self-identified as AI in the T1000 study were matched with NHW individuals from the original sample on sociodemographic variables (i.e. sex, age, income, education and WRAT reading score), which have been shown to be associated with differential risk for STB and SUD, as well as differential fMRI metrics of executive function (Graham *et al.*, 2010; Patrick *et al.*, 2012; Page *et al.*, 2014). Due to its role as a psychological risk factor, trauma exposure (i.e. CTQ and TES) was also included in the matching procedure. The resulting 1-to-1 subsample of participants was across two groups, AI and NHW. Propensity matching is employed to address confounding in the context of observational studies (Austin, 2011) often results in balance across comparison groups with respect to variables included in the matching; however, this result should be verified analytically (Ohlsson and Kendler, 2020). Thus, results of the procedures with respect to matching variables can be seen in Table 2. To evaluate the relative incidence of STB and SUD, chi-square analyses were conducted across the two groups on each variable separately.

**Table 2.** Propensity matched verification in matched American Indian (AI) and non-Hispanic White (NHW) participants

	AI (*n* = 76)		NHW (*n* = 76)				
	*M*	*SD*	*M*	*SD*	*t*	*df*	*p*
Age (years)	33.36	9.46	34.48	10	−0.56	150	0.58
WRAT	60.25	6.24	60.66	6.43	0.40	150	0.69
Income ($)	25 221.45	26 942.43	22 968.00	27 369.13	−0.51	150	0.61
TES occurrence (total)	3.88	3.25	4.13	3.62	0.45	148	0.65
TES intensity (worst)	10.38	7.69	10.87	6.89	0.41	148	0.68
CTQ denial	8.25	3.43	7.99	3.11	−0.50	149	0.62
CTQ emotional abuse	10.45	5.17	10.99	5.06	0.65	150	0.52
CTQ emotional neglect	11.08	5.28	11.58	4.87	0.61	149	0.54
CTQ physical abuse	8.88	4.02	8.99	4.33	0.16	149	0.88
CTQ physical neglect	7.97	3.28	7.96	3.57	−0.02	149	0.98
	Frequency		Frequency				
Education (categories)					*Χ* ^2^ = 6.85, *p *= 0.08		
Less than high school	11 (14.74%)		6 (7.90%)				
High school or GED	20 (26.32%)		17 (22.37%)				
Some college	21 (27.63%)		36 (47.37%)				
College degree or higher	24 (31.58%)		17 (22.37%)				
Sex (female)	46 (60.53%)		46(60.53%)		*n/a*		

Note: WRAT = Wide Ranging Achievement Test; TES = Traumatic Experiences Scale; CTQ =** **Childhood Trauma Questionnaire.

#### Inhibitory control

The second aim of the study is to determine if within the AI sub-sample, there is an association between neural indicators of inhibitory control and STB or SUD. Welch’s two sample *t*-tests, which are robust to the assumption of homogeneity of variance (Zimmerman, 2004; Field *et al.*, 2012), were used to examine mean PSC differences within *a priori* ROIs (i.e. dlPFC [3 bilateral ROIs], IFG [2 bilateral ROIs]) using BNA defined ROIs (see Table S3, Figure S4) to compare individuals with and without current STB and SUD. Exploratory whole brain analyses were also conducted (see supplementary material).

## Results

### Incidence comparisons

**Fig. 1.**
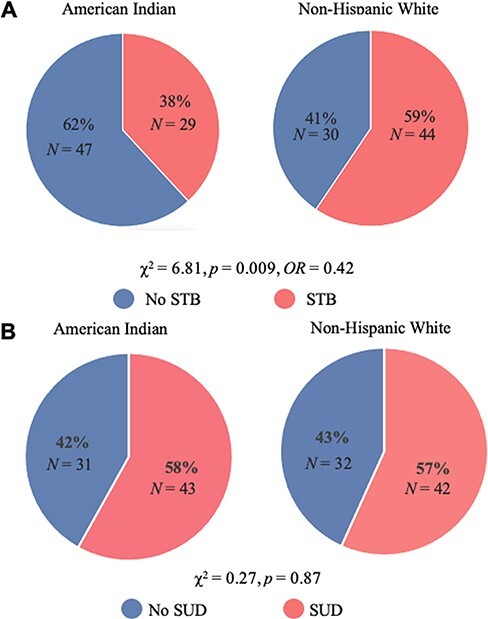
Pie charts displaying incidence rates comparison between AI and NHW in the matched sample.

*Note*: STB = suicidal thoughts and behaviors; SUD = substance use disorder.

The groups did not differ on matching variables following the propensity matching procedures (see Table 2). With respect to STB, individuals in the AI group displayed a lower incidence when compared with the matched sample of NHW (*Χ*^2^ = 6.81, *P *= 0.009, *OR *= 0.42; Figure 1A). Regarding SUD incidence, there were no differences between the AI group and NHW group (*Χ*^2^ = 0.028, *P *= 0.87; Figure 1B). Chi-squared analysis in the unmatched samples can be found in supplementary material.

### Neuroimaging

Among the AI sample, individuals without STB, relative to individuals with STB, demonstrated increased BOLD signal activation in the left dlPFC (BNA-ROI-19: *t*(49.06) = 2.44, *P* = 0.02 Hedge’s *g = *0.60 CI_95_ = [0.09, 1.11]; BNA-ROI-21: *t*(57.71) = 2.15, *P* = 0.04, Hedge’s *g = *0.50 CI_95_ = [−0.01, 1.01]); right dlPFC (BNA-ROI-20: *t*(51.9) = 2.06, *P* = 0.04, Hedge’s *g = *0.50 CI_95_ = [−0.01, 1.01]) and left IFG (BNA-ROI-31: *t*(45.393) = 2.22, *P *= 0.03, Hedge’s *g = *0.56 CI_95_ = [0.05, 1.07]) during hard *vs* easy contrast of the SST. No other BNA ROIs were significantly different between STB groups (*P’*s = 0.06–0.22).

Interestingly, with respect to SUD, individuals without SUD demonstrated significantly lower BOLD signal response in the left IFG (BNA-ROI-31: *t*(57.36) = −2.00, *P* = 0.05 Hedge’s *g = *−1.581 CI_95_= [−1.96, −1.20]) and left dlPFC (BNA-ROI-19: *t*(57.12) = −2.56, *P* = 0.01 Hedge’s *g = *−1.582 CI_95_= [−1.96, −1.20]; BNA-ROI-21: *t*(58.53) = −2.1, *P* = 0.03 Hedge’s *g = *−1.583 CI_95_= [−1.96, −1.20]); no other BNA ROIs showed significant differences between SUD groups (*P*’s = 0.06–0.49). See supplement for exploratory whole-brain linear mixed effect models. Of note, the dlPFC group differences based on STB were also significant in whole-brain analyses (voxel-wise threshold of *P *< 0.005; cluster corrected at *P *< 0.05 using a threshold of 83 voxels; Figure S2).

## Discussion

This study addressed two questions: (i) to determine the incidence of STB and SUD in AI individuals, after accounting for sociodemographic variables and trauma exposure and (ii) to examine possible neural mechanisms by examining associations of STB and SUD with neural markers of inhibitory control in AI participants. There were three main results. First, after accounting for sociodemographic variables as well as trauma exposure as a risk factor, AI demonstrated lower incidence of STB and equivalent rates of SUD relative to a matched NHW sample. Second, AI participants with no STB relative to those with reported STB showed greater activity in executive control regions during the SST. Third, AI individuals with a history of SUD showed higher activation in executive control regions than those with no history. These findings are an important addition to this literature as AIs have displayed the largest increase in suicide rates of any ethnic group in the recent decade (Curtin and Hedegaard, 2019) and have displayed high prevalence of SUD (Beals, 2005; Brave Heart *et al.*, 2016). These data extend previous findings that indicate the high level of risk (i.e. trauma exposure) accounting for inflated prevalence of PTSD in a Southwestern AI tribe (Robin *et al.*, 1997). Taken together, this body of evidence supports two critical implications: (i) there is an extremely high risk burden in AI communities (Les Whitbeck *et al.*, 2004; Alcántara and Gone, 2007) and (ii) when accounting for risk level and socioeconomic variables, AI individuals may have important protective factors *against* poor mental health (i.e. STB and SUD). This is consistent with previous research demonstrating high incidence of positive mental health among two AI communities despite disproportionately high stressors (Kading *et al.*, 2015).

The current investigation also explored the potential for neural factors that may be protective among the AI sub-sample. Results from the fMRI BOLD signal data indicate that individuals with no STB demonstrated greater activation in brain regions associated with executive control (i.e. dlPFC and IFG; Matthews *et al.*, 2005) during a response inhibition task than those at risk. Extant literature supports the beneficial role of inhibitory control on emotion regulation (Diamond, 2013) and documents low levels of inhibitory control across multiple psychopathologies (McTeague *et al.*, 2016) and suicidality (Jollant *et al.*, 2011). Thus, current results extend this literature by demonstrating that inhibitory control is associated with lower STB among AIs. Although, the potential adaptive effects of inhibitory control are not unique to AIs, this study provides initial evidence that inhibitory control is relevant for clinical neuroscience research among AIs and future work in this area should aim to identify factors that support optimal inhibitory control among AIs specifically.

This activation pattern was in the opposite direction for individuals with SUD relative to no history of SUD. Thus, inhibitory control-related BOLD signal was greater in AI individuals with relative to those without SUD. Notably, inhibitory control disruptions are a key risk factor for SUD (Heitzeg *et al.*, 2015). However findings regarding fMRI activation are mixed with respect to dlPFC and IFG BOLD signal differences in substance use disorder (Zilverstand *et al.*, 2018; Hildebrandt *et al.*, 2021; Le *et al.*, 2021). Direction of activation differences (hypo/hyperactivation) may be dependent on substance type and current use *vs* abstinence (see: Le *et al.*, 2021); furthermore, a recent systematic review indicates that degree of substance use and substance related difficulties may play a key role in differential neural activity associated with response inhibition (Hildebrandt *et al.*, 2021). Importantly, current data include substance use disorder diagnosis within the past year and participants report a range of substance preferences (see Table S1). Our data suggest that AI individuals with substance use disorder show greater activation in prefrontal cortical regions during inhibition. However, more work is needed to delineate the nuance in the relationship of substance type and use *vs* abstinence and neural markers of inhibition broadly and especially among AI populations.

These findings point to the intriguing possibility of culturally based resilience to mental health problems among AI individuals that may be evidenced by adaptive neurocognitive functioning. Recent research has identified specific cultural influences among AIs (i.e. enculturation, spirituality and traditional healing) and differences in social support (i.e. extended family networks and tribal connection) that may be protective against poor mental health (i.e. SUD and suicidality; Fleming and Ledogar, 2008) and predictive of well-being (LaFromboise *et al.*, 2006; Kirmayer *et al.*, 2011). There are multiple factors of particular salience to AI individuals (e.g. enculturation and spirituality) and communities (e.g. social connectedness; LaFromboise *et al.*, 2006; Fleming and Ledogar, 2008; Kirmayer *et al.*, 2011) that may enhance inhibitory control (Tsethlikai, 2015). Theoretical work indicates that sociocultural connections convey the resilience against major life stressors (Doane *et al.*, 2012) and play an important role in the development of the ability to cognitively manipulate information (Dunbar, 2003). Specifically, rooted in evolutionary theory, the social brain hypothesis posits that social group complexity in humans is closely evolutionarily interconnected with cognitive control processes, perhaps given the use of such processes for tracking and navigating the structure of their social networks and relationships therein (Dunbar, 2003; Stout, 2010). Future work is needed to establish the potential relationships between cultural factors and adaptive neurocognitive function among AI populations.

Current findings should be contextualized within notable limitations to the data. The cross-sectional nature of these data precludes the ability to make causal or directional inferences regarding identified relationships. Due to the novelty of the current approach, we chose to focus analyses on mental health difficulties noted as particularly relevant for AI populations (Beals, 2005; Brave Heart *et al.*, 2016; Curtin and Hedegaard, 2019) and physical and mental health comorbidities were not evaluated in the present analysis. Notably, suicidality was indexed based on the MINI clinical interview suicidality module rather than previous suicidal behaviors. However, this module has been shown to be predictive of suicidal behaviors (Roaldset *et al.*, 2012). Although dichotomizing the data into risk and absence of reported risk eliminates some granularity in the measurement of suicide risk, it is a reasonable first approximation of suicidality in the current sample. Future investigations are needed to examine cognitive control suicide related ideation and behaviors among AI, as the current study was not designed or powered to examine suicide with a high degree of specificity. Furthermore, this examination comprises secondary data analysis that was not designed to address the particular risk and protective factors among AI. Thus, there was no data collected regarding specific risk factors (e.g. historical trauma and discrimination) and/or cultural protective factors (e.g. familial structures, social support and traditional culture engagement) that may be present within this sample. Notably in the unmatched samples, AI demonstrated lower STB and increased SUD incidence relative to NHW; however, this does not preclude the examination of neural mechanisms of protective effects in the current sample. Importantly, there is substantial heterogeneity among AI peoples with over 573 federally recognized tribes in the USA and variability in whether individuals live on or off reservations and in urban or rural settings (Weaver, 2012); the current analyses cannot account for the influence of heterogeneity among the AI sample. Although the current investigation focused on sociodemographic factors and trauma exposure as risk factors for STB, it is important to note that other factors also play a role in risk for STB (e.g. depression and other psychopathology symptoms). Furthermore, mechanistic research in STB among AI populations is very limited; thus, the field would benefit from future investigations examining mechanisms of the relationship between symptoms of psychopathology and STB among AIs. Additionally, inhibitory control only represents one potential mechanism of protective factors against STB and SUD. Future research is needed to examine culturally specific variables that may convey protective effects as well as a broader range of potential neural mechanisms.

## Conclusions

The current findings indicate that, when accounting for sociodemographic and psychological risk factors (i.e. trauma exposure), STB are lower and SUD is not different among a sample of AI compared with NHWs recruited from the general community. Furthermore, examination of fMRI BOLD signal activation during SST revealed that AI individuals without STB displayed greater activation in prefrontal cortical regions associated with executive control (e.g. dlPFC and IFG) than AI with STB. In sum, contrary to conclusions drawn from raw prevalence statistics (Curtin and Hedegaard, 2019), contextualization of prevalence of STB indicate there may be protective factors against STB among AI. Furthermore, inhibitory control may represent a potential mechanistic aspect of adaptive neural functioning relevant for AI mental health research, thus adding to a burgeoning literature on cultural factors that promote mental health among AIs (Kading *et al.*, 2015).

## Supplementary Material

nsac045_Supp

## Footnotes

1 It is important to note that terms such as American Indian/Alaska Native (AIAN), Native, Indigenous, First Peoples, have often been used interchangeably and it is most respectful to use terminology individuals and communities prefer. For the current study, we use the term AI to refer to individuals from heterogenous Native tribes represented in northeastern Oklahoma.
