# Supplementary material for: Neural processes of inhibitory control in American Indian peoples are associated with reduced mental health problems
Source: Soc Cogn Affect Neurosci. 2022 Jul 8;18(1):nsac045. doi: 10.1093/scan/nsac045 (PMC9949499; doi:10.1093/scan/nsac045)
Supplement: nsac045_Supp [file nsac045_supp.zip › Supplemental material.docx]

**Supplemental material**

**Neural processes underlying inhibitory control in American Indian peoples is associated with reduced mental health problems.**

Evan J. White, Ph.D., Mara J. Demuth, M.A., Mariah Nacke, B.S., Namik Kirlic, Ph.D., Rayus Kuplicki^,^ Ph.D., Phillip A. Spechler, Ph.D., Timothy J. McDermott, M.A., Danielle C. DeVille, M.A., T1000 investigators, Jennifer L. Stewart, Ph.D., John Lowe, R.N., Ph.D.**,** Martin P. Paulus, M.D. & Robin L. Aupperle, Ph.D.

**Prevalence analysis in unmatched samples.**

With respect to STB individuals in the AI group displayed a lower rate as compared to NHWs (*Χ* ^2^= 5.74, *p* = 0.02, *OR* = 1.77). Regarding SUD, the AI group displayed a higher rate as compared to NHWs (*Χ* ^2^ = 11.33, *p* < 0.001, *OR =* 0.44). It is important to note that these comparisons do not account for potential sociodemographic and/ or psychological risk factors that are present between these groups both in observed and potentially unobserved variables. Thus, examination of the propensity matched samples in the main text offers a more appropriate comparison.

**Table S1.** *Frequency of drug of choice responses reported in matched samples*

| CDDR drug of choice | Non-Hispanic White (*n*) | American Indian (*n*) |
| --- | --- | --- |
| Amphetamines | 15 | 18 |
| Alcohol | 26 | 29 |
| Benzodiazepines | 0 | 3 |
| Cocaine | 1 | 0 |
| Cannabis | 6 | 4 |
| None | 20 | 14 |
| Opiates | 8 | 8 |

*Note:* CDDR = Customary Drinking and Drug use Record

| **Diagnostic method** | **Number of AI subjects** |
| --- | --- |
| MINI | 24 |
| Tulsa Life Chart | 3 |
| MINI & Tulsa Life Chart | 8 |
| No SUD | 33 |

***Table S2.*** Number of AI subjects in SUD group by method of assessment

*Note:* AI = American Indian; SUD = substance use disorder; MINI = MINI International Neuropsychiatric Interview

| **Table S3.** *Brainnetome ROIs used in current analysis labels, anatomical descriptors, and coordinates of ROI center mass. adapted from table reported in the cytoarchitectural atlas (Fan et al., 2016)* | | | | | | |
| --- | --- | --- | --- | --- | --- | --- |
| **Gyrus** | **Left and Right Hemisphere** | **Label ID.L** | **Label ID.R** | **Anatomical and modified Cyto-architectonic descriptions** | **lh.MNI(X,Y,Z)** | **rh.MNI(X,Y,Z)** |
| **MFG, Middle Frontal Gyrus** | MFG_L(R)_7_1 | 15 | 16 | *A9/46d, dorsal area 9/46* | -27, 43, 31 | 30, 37, 36 |
|  | MFG_L(R)_7_3 | 19 | 20 | *A46, area 46* | -28, 56, 12 | 25, 55, 17 |
|  | MFG_L(R)_7_4 | 21 | 22 | *A9/46v, ventral area 9/46* | -41, 41, 16 | 42, 44, 14 |
| **IFG, Inferior Frontal Gyrus** | IFG_L(R)_6_2 | 31 | 32 | *IFS, inferior frontal sulcus* | -47, 32, 14 | 48, 35, 13 |
|  | IFG_L(R)_6_3 | 33 | 34 | *A45c, caudal area 45* | -53, 23, 11 | 54, 24, 12 |
|  | IFG_L(R)_6_5 | 37 | 38 | *A44op, opercular area 44* | -39, 23, 4 | 42, 22, 3 |

**Figure S1.** *Hard versus easy trial contrast from the stop-signal paradigm in the parent sample.*

*
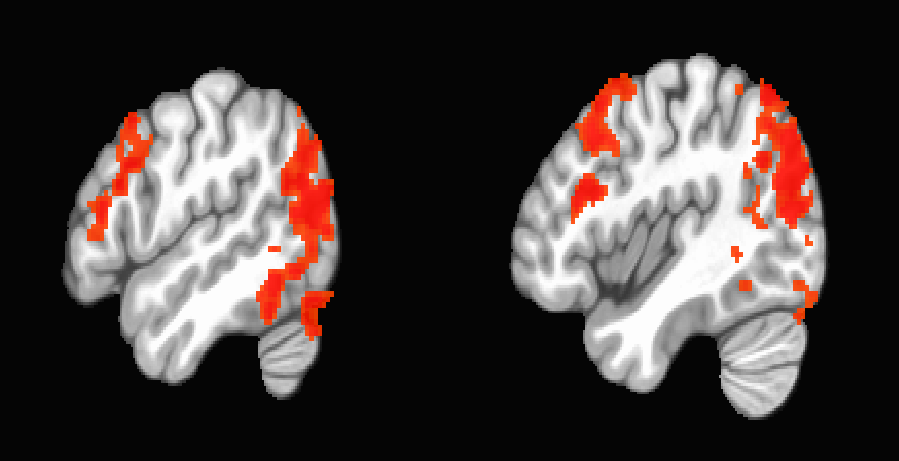
*

*Note.* Red indicates more activation. Voxelwise significant threshold at *p* < 1x 10^-5^

**Table S4.** *Cluster location descriptions and mean test statistic for Figure S1*

| Number of Voxels | Center of Mass (X,Y,Z) | Mean *Z*-statistic |
| --- | --- | --- |
| 27057 | 0.8, 71.8, 17.7 | 5.39 |
| 2173 | 34.4, -20.8, 47.5 | 5.01 |
| 573 | -26.9, 18.9, -15.6 | 5.27 |
| 496 | -15.7, -25.8, -6.9 | 4.75 |
| 353 | -24.9, -27.4, 48 | 4.89 |
| 318 | 3.5, -61.1, -5.8 | 4.91 |
| 279 | -36.4, 23.7, 19.7 | 4.89 |
| 181 | -48.5, -31.5, 16.3 | 4.71 |
| 151 | 31.1, -35.4, -12.4 | 5.00 |
| 119 | 43.5, 13.9, 35.4 | 5.00 |
| 112 | 20.9, -66.7, 10 | 4.82 |
| 104 | -32.9, -23, 0.3 | -4.87 |
| 62 | -20.2, -11.6, 26.9 | 4.89 |
| 60 | -27.6, 36.8, 3.3 | 4.77 |
| 47 | 0.7, -69.6, 18.3 | 4.68 |
| 47 | 1.2, 12.2, 19 | 4.68 |
| 47 | -14.5, 11.4, 26.4 | 4.77 |
| 46 | 4, 33.5, -3.6 | 5.00 |
| 45 | -6.5, 31.6, -3.4 | 4.68 |
| 45 | 60.4, 11.4, 2.1 | 4.68 |
| 44 | -34.9, 5.7, 67.8 | 4.73 |
| 43 | -58, 3.2, -15.7 | 4.74 |

**Exploratory whole brain analyses**

Linear-mixed effect modeling on whole brain data was used to model the interaction of hard vs. easy contrasts in the stop signal data and SR as well as SUD separately. The results were cluster corrected with auto-correlated function in AFNI(Cox, 2012) using 3dClustSim and limited to a significance threshold of *p <* .005. For SR one cluster survived cluster correction and thresholding (Figure S2.) comprising 83 voxels with a center of mass at the following location (-27.0, -45, 21.). For SR one cluster survived cluster correction and thresholding (Figure S2.)

**Figure S2.** *Whole brain linear mixed effect model interaction between suicide risk and hard versus easy stop-signal trials among the AI subsample.*


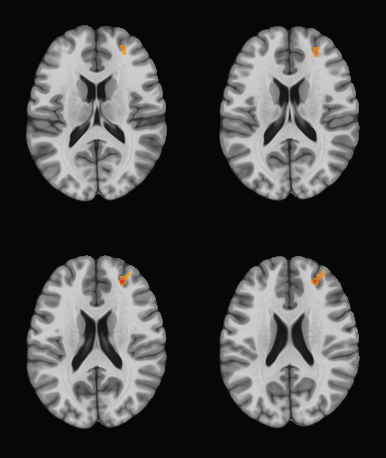

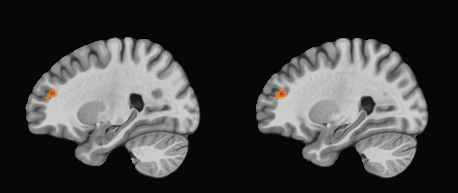

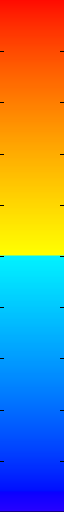


18.48

14.27

10.65

7.03

3.26

0

R

L

**Figure S3.** *Whole brain linear mixed effect model interaction between substance use disorder*


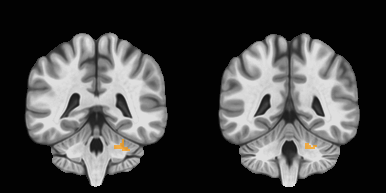


18.48


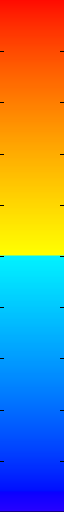


14.27

10.65

7.03

0

3.26

L

R


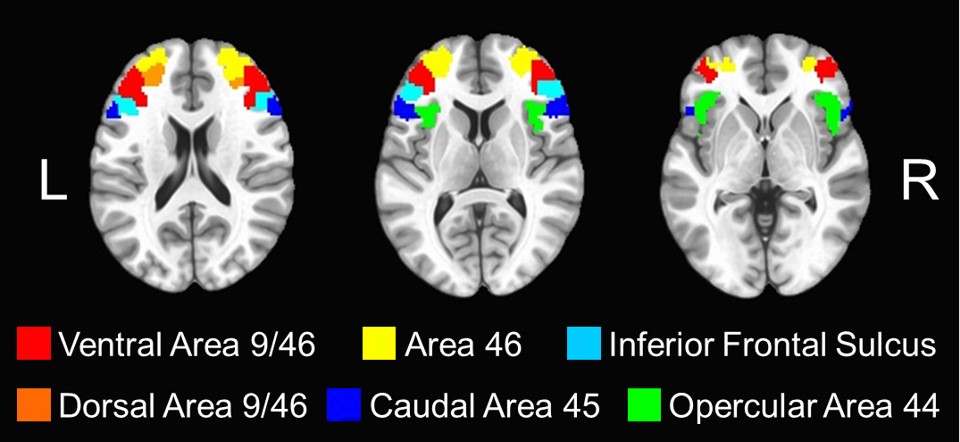
**Figure S4.** Brainnetome Atlas regions of interest (dlPFC and IFG)
